# Genetic Parameters and Weighted Single-Step Genome-Wide Association Studies of Fertility Traits in Chinese Holstein †

**DOI:** 10.3390/ani16111622

**Published:** 2026-05-26

**Authors:** Shanshan Li, Ao Wang, Yao Chang, Qingxia Yan, Hailiang Zhang, Shiyu Hou, Gang Guo, Yachun Wang

**Affiliations:** 1 State Key Laboratory of Animal Biotech Breeding, National Engineering Laboratory for Animal Breeding, Laboratory of Animal Genetics, Breeding, and Reproduction, College of Animal Science and Technology, China Agricultural University, Beijing 100193, China; lishsh@cau.edu.cn (S.L.); lxxkwa@163.com (A.W.); o188chang@163.com (Y.C.); zhl108@cau.edu.cn (H.Z.); 2 Dairy Association of China, Beijing 100193, China; qingxiayan1982@163.com; 3 Anshan Hengli Dairy Farm, Anshan 114200, China; hsy3569999@163.com; 4 Beijing Sunlon Livestock Development Company Limited, Beijing 100029, China; guogang2180@126.com

**Keywords:** Chinese Holstein, weighted single-step genome-wide association study, fertility trait, QTL region, candidate genes

## Abstract

Fertility traits are core functional traits in dairy cattle, yet their low heritability makes traditional phenotypic selection inefficient. Weighted single-step GWAS (WssGWAS) improves detection power by integrating pedigree, phenotype, and genotype data. This study aimed to estimate genetic parameters and identify genomic regions associated with eight fertility traits in Chinese Holstein cattle using pedigree, phenotype, and genotype data. Genetic parameters were estimated via pedigree-based BLUP (PBLUP) and ssGBLUP, and a WssGWAS was performed to detect QTL. A total of 23,771 cattle were genotyped using liquid-phase chips and 150 K chips; after imputation and quality control, 13,690 individuals and 103,262 SNPs were retained. The eight traits comprised heifer traits (AFS, AFC, NS_H, and IFL_H) and cow traits (ICF, CI, NS_C, and IFL_C). The findings indicate that while most fertility traits exhibit low heritability (ranging from 0.024 to 0.085), AFS displays high heritability (0.390). WssGWAS identified 162 QTL intervals and 645 candidate genes, including *OVOS2*, *PMEPA1*, *TEPP*, and the *RAG* family, which are closely associated with reproductive function. These findings provide a foundation for future research into the genetic mechanisms of fertility and support improved genomic predictions in Chinese Holstein cattle.

## Simple Summary

Fertility is essential for dairy cows, but intensive selection for higher milk yield has led to poorer reproductive performance due to adverse genetic correlations between milk production and fertility traits. Given the low heritability of fertility traits, improving the accuracy of genomic prediction for these traits is highly important. This study investigated eight fertility traits in Chinese Holsteins, including heifer traits such as age at first service and cow traits like calving interval. A total of 13,690 individuals with 103,262 genetic loci were analyzed after quality control and genotype imputation. Heritability of these traits ranged from 0.024 ± 0.002 to 0.390 ± 0.008. Using weighted single-step genome-wide association studies (WssGWAS), 645 candidate genes were identified, among which *OVOS2*, *PMEPA1*, *TEPP*, and the *RAG* family were closely related to fertility. These results help clarify the genetic basis of fertility traits and support accurate genomic selection for reproductive performance in Chinese Holstein cattle.

## 1. Introduction

Fertility traits are core functional traits of Holstein cattle, directly affecting the reproductive efficiency, herd renewal rate, and overall profitability of the dairy industry. However, Holstein fertility traits (e.g., age at first breeding, pregnancy rate, calving interval) are typical complex traits with low heritability, generally ranging from 0.03 to 0.15 [1,2]. The low heritability and susceptibility to environmental factors make traditional phenotypic selection inefficient for genetic improvement of these traits, resulting in slow genetic progress in Holstein reproductive performance. Therefore, exploring efficient genetic analysis methods to dissect the molecular mechanisms underlying fertility traits is crucial for accelerating the genetic improvement of Holstein cattle and promoting the sustainable development of the dairy industry.

Genome-wide association studies (GWAS) have emerged as a potent methodology for identifying genetic variations associated with complex traits across livestock, plants, humans, and model organisms [3]. To date, multiple GWAS have identified quantitative trait loci (QTLs) and genetic variants associated with dairy cattle fertility traits, including calving interval, number of services, and interval from calving to first service, providing valuable insights into the genetic basis of these traits [4,5]. However, conventional GWAS approaches rely exclusively on genotyped individuals with matched phenotypic records, ignoring pedigree information from non-genotyped animals, which leads to substantial information loss and low detection power, especially for low-heritability traits like dairy cattle fertility [6,7]. To address this limitation, the weighted single-step GWAS (WssGWAS) method was developed, which simultaneously integrates pedigree, phenotypic, and genomic data in a single analysis framework [8]. WssGWAS enhances QTL detection in low-heritability traits by integrating genotype, pedigree, and phenotype in a single step, avoiding information loss typical of multi-step analyses [9,10]. It improves statistical power and optimizes SNP effect estimation through iterative weighting, increasing sensitivity to detect loci with small effects [8,11]. For instance, Tenghe, Bouwman, Berglund, Strandberg, de Koning and Veerkamp [2] used a mixed model to analyze endocrine and traditional fertility traits and found that their heritability was only 0.03–0.15, while the single-step method could improve the detection sensitivity through weighted labeling effect. This method has been successfully applied in the genetic dissection of fertility traits in dairy cattle and other livestock species in recent studies [12,13].

In recent years, the liquid chip based on genotyping by target sequencing (GBTS) technology has emerged as a cost-effective and flexible alternative to traditional solid-phase SNP chips [14]. Compared with solid chips, GBTS-based liquid chips offer higher design flexibility, lower per-sample cost, and the ability to target specific genomic regions associated with traits of interest, while maintaining high genotyping accuracy [15,16]. These advantages make liquid chips particularly suitable for large-scale population genomic analyses in livestock breeding, as they can support high-throughput genotyping at a reduced cost without compromising analytical power [17]. Previous research has shown that liquid chips can achieve comparable or even superior performance in genomic prediction and GWAS for economically significant traits in livestock [18,19]. However, few studies have applied liquid chips combined with WssGWAS to analyze the genetic basis of fertility traits in Chinese Holstein cattle, and the performance of liquid chips in this context remains to be fully evaluated.

Therefore, this study aimed to (1) estimate the variance components, heritability, and repeatability of eight fertility traits in a large Chinese Holstein population using pedigree, phenotypic, and genotypic data from both liquid-phase and 150 K solid chips, and (2) to identify significant genomic regions and candidate genes associated with these fertility traits via WssGWAS. The findings of this study are expected to lay a theoretical foundation for the genomic breeding of dairy cattle fertility traits and to provide technical support for the genetic improvement of Chinese Holstein cattle.

## 2. Materials and Methods

### 2.1. Ethics Statement

The whole procedure for blood sample collection was carried out in strict accordance with the protocol approved by the Animal Care and Use Committee of China Agricultural University (protocol code AW31013202-1-2 and 13 October 2023 of approval).

### 2.2. Phenotypic and Pedigree Data

Phenotypic data were extracted from event records exported from the Afimilk management system. The data format for genetic evaluation in Beijing was updated in 2019; to ensure consistency across the entire dataset, all event records from 2019 onwards were standardized to the pre-2019 format, and fields exclusive to the post-2019 format were excluded. Records of birth, calving, insemination, and pregnancy check information were collected in 56 herds located in Beijing, China from 1990 to 2022. A total of 419,870 birth records, 523,053 calving records, 1,796,572 insemination records and 3,610,399 pregnancy check records were available. Cows on these farms were housed in free-stall barns and total mixed ration was delivered daily. All 56 herds were located in the Beijing metropolitan area, within the North China Plain, characterized by a temperate continental monsoon climate with similar topography, ambient temperature patterns, and humidity conditions. The concentrated geographical distribution ensures that macro-environmental variability across herds was limited. Detailed descriptions of event types, field definitions, and calculation methods for the eight fertility traits are provided in Supplemental Materials and Methods (Supplementary_M&M). A total of eight traits were analyzed in this study for heifers and cows. The heifer traits included age at first service in heifer (AFS; in days), age at first calving in heifer (AFC; in days), number of services for heifer (NS_H), and interval from first to last inseminations in heifer (IFL_H; in days). The cow traits included interval from calving to first service (ICF; in days), calving interval in cow (CI; in days), number of services for cows (NS_C) and interval from first to last inseminations in cows (IFL_C; in days). The IFL_H and IFL_C were 0 when a heifer/cow was pregnant after the first service. To address the data censoring caused by culling and incomplete service events, only cows born between 1999 and 2022 and the service and calving events occurring from 2000 to 2022 were retained. The valid insemination records in a lactation were identified by a positive pregnancy test at the last service or confirmed subsequent calving events. Further criteria for the data editing included AFS between 270 and 900 days, AFC between 500 and 1100 days, CI between 280 and 600 days, NS_H and NS_C between 1 and 10 times, IFL_H and IFL_C at 0 days or between 19 and 355 days, and ICF between 20 and 230 days. Animals which changed herds during the analyzed period were excluded. For cows that were culled or exited the herd before a confirmed pregnancy was recorded, the last incomplete service record was excluded from the analysis, as the pregnancy outcome could not be verified. Consequently, only lactations with a confirmed pregnancy outcome (positive pregnancy check or subsequent calving) were retained. After quality control, the number of records for each fertility trait was performed, as shown in Table 1. The final dataset included cows ranging from parity 1 to 13. Individual health records and body condition scores were not systematically recorded across all farms and time periods, and therefore were not available for inclusion in the statistical models The pedigree information was obtained from birth and calving records. Each animal with phenotypic records was tracked back as many generations as possible. The final pedigree contained 521,222 animals born from 1907 to 2020, among which there were 13,305 sires.

### 2.3. Genotypic Data

#### 2.3.1. Imputation

A total of 20,294 cows and 3477 bulls were genotyped. Among all animals, 5089 cows and 81 bulls were genotyped with the GeneSeek Bovine 150 K chip (Illumina Bovine 150 K SNP, Illumina Inc., San Diego, CA, USA); 5545 cows were assayed with liquid-phase chips, and the remaining cattle were assayed with the 50 K chip (Illumina Bovine 50 K SNP, Illumina Inc., San Diego, CA, USA). The SNPs from the 150 K and 50 K chips (originally based on UMD 3.1) were converted to ARS_UCD 1.2 using UCSC liftover command (https://genome.ucsc.edu/cgi-bin/hgLiftOver, accessed on 10 April 2026), which was used in the liquid chips. Subsequently, phasing and genotypic imputation were performed using Beagle v5.4 [20,21]. The process followed two steps: (1) using the 150 K as the reference panel, the 50 K and liquid chips are imputed to 150 K density; (2) using the liquid chip as the reference panel, the 150 K chips are imputed to the density of the liquid chip. Notably, the 7518 InDel variants present on the liquid-phase chip were excluded prior to imputation. This decision was primarily driven by the absence of matching InDels on the 150 K and 50 K chips, which precluded reliable cross-platform imputation and downstream harmonization. Moreover, the genotyping accuracy for small insertions and deletions is generally lower than for SNPs in array-based platforms, and the statistical models used in subsequent analyses (e.g., ssGBLUP, WssGWAS) assume biallelic SNP markers. Retaining InDels would have introduced a heterogeneous marker class with uncertain calling and imputation quality, potentially biasing SNP effect estimation and genomic prediction. Therefore, only high-quality, common biallelic SNPs shared across platforms were retained to ensure a robust and uniformly processed marker set. Imputation accuracy was assessed using the mean dosage R^2^ (DR^2^) per SNP, as reported by Beagle v5.4. Across all chromosomes, the mean DR^2^ was 96.34% (SD = 0.05) for imputation from 50 K to 150 K, 96.95% (SD = 0.04) for liquid-phase chip to 150 K, 95.26% (SD = 0.04) for 150 K to liquid-phase chip. Less than 2% of SNPs had a DR^2^ < 0.80, and these low-confidence variants were excluded prior to downstream analyses. To validate imputation reliability, we masked 5% of genotypes from each of the 150 K and liquid-phase chip reference panels and compared the imputed genotypes to the original calls. The correlation between true and imputed allele dosages was 99.22% for 50 K to 150 K, 99.61% to liquid-phased chip to 150 K, and 99.14% for 150 K to liquid-phase chip, confirming high imputation fidelity.

#### 2.3.2. Quality Control

**Figure 1:**
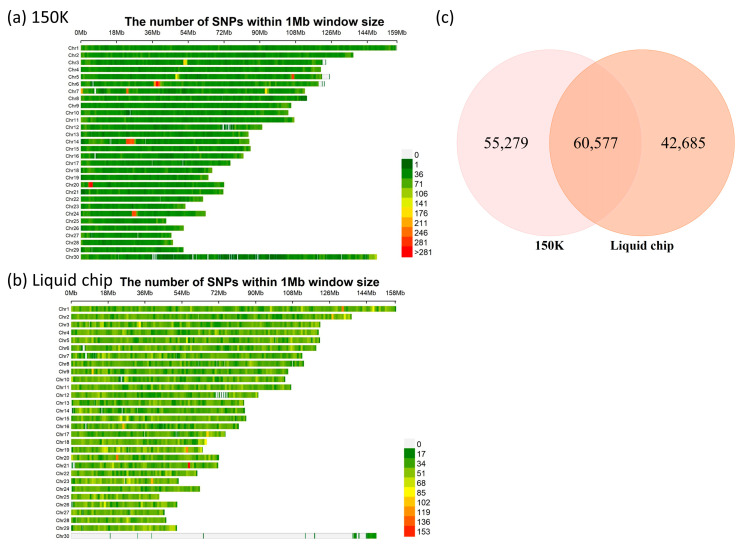
The SNP density plot and Venn plot. (**a**) SNP density plot representing the number of quality-passed SNPs within each 1 Mbp window size for 150 K chip. (**b**) SNP density plot representing the number of quality-passed SNPs within each 1 Mbp window size for liquid chip. (**c**) Venn plot of SNPs between the liquid chip and the 150 K chips.

Genomic data were quality-controlled using PLINK v1.9 [22] software with the following criteria: (1) Individual genotype call rate greater than 90%; (2) minor allele frequency (MAF) greater than 0.05; and (3) *p* value for Hardy–Weinberg equilibrium greater than 10 × 10^−6^. Finally, a total of 13,690 individuals with 115,856 SNPs from the 150 K chip and 103,262 SNPs from the liquid chip were retained for further analysis. A relatively stringent MAF threshold of 0.05 was applied to minimize spurious associations driven by rare variants that are particularly susceptible to genotyping errors and imputation uncertainty in our sample size. This threshold is consistent with previous GWAS studies in dairy cattle with similar cohort sizes [23]. The SNP density of the 150 K chip and the liquid chip after quality control (each 1 Mbp window size) are shown in Figure 1a,b, which were generated by the ‘CMplot’ (v 4.5.1) package in R-4.3.3 [24]. Figure 1c presents the number and overlap of SNPs between the 150 K chip and the liquid chip. A total of 60,577 identical loci were identified, accounting for 52.29% and 58.66% of the total number of SNPs on the 150 K chip and liquid chip, respectively.

### 2.4. Statistical Analysis

To better understand the genetic background of these traits, we employed two methods, PBLUP and ssGBLUP, to estimate genetic variance and to calculate heritability as well as repeatability. Subsequently, for the purpose of enabling a more effective comparison of the heritability and repeatability of the two chips, the two chips were separately utilized to establish the genetic relationship for ssGBLUP (ssGBLUP_150K and ssGBLUP_LC).

The genetic parameters for the heifer traits (AFS, AFC, IFL_H and NS_H) were estimated by a single-trait animal model, as shown in **Model (1)**. The genetic parameters for the cow fertility traits (ICF, IFL_C, NS_C, CI) were estimated by a single-trait repeatability model, as shown in **Model (2)**. The models can be described as follows:

(1) y=Xb+Za+e

(2) y=Xb+Za+Wpe+e

where **y** represents the phenotypic value vector; **X**, **Z**, and **W** denote the incidence matrices for fixed effects, additive genetic effects, and permanent environmental effects, respectively; **b** represents the fixed effect vector (the fixed effects for each trait are presented in Table 2); a represents a vector of additive genetic effects; pe represents a vector of random permanent environmental effects; and e represents the vector of random residuals. It was assumed that $a\sim N(0,K\sigma_{a}^{2})$, $pe\sim N(0,I\sigma_{pe}^{2})$, and $e\sim N(0,I\sigma_{e}^{2})$, where the **K** matrix is the **A** (relationship matrix based on pedigree information) or **H** (relationship matrix based on genotypic and pedigree information) matrix, $\sigma_{a}^{2}$ is the additive genetic variance, I is an identity matrix, $\sigma_{pe}^{2}$ is the permanent environmental variance, and $\sigma_{e}^{2}$ is the residual variance. The inverse of **H** was calculated as follows [25]:

$$H^{-1}=A^{-1}+\left[\begin{array}{cc} 0 & 0\\ 0 & G^{-1}-A_{22}^{-1} \end{array}\right]$$

where **G** is the genomic relationship matrix based on genotype information, and **A_22_** is the numerator relationship matrix for genotyped animals. The **G** matrix was computed as follows [26]:

$$G=\frac{ZDZ^{\prime }}{\sum_{i=1}^{M}2p_{i}(1-p_{i})}$$

where **Z** is a matrix of gene content adjusted for allele frequencies (0, 1, or 2 for aa, Aa, and AA, respectively); **D** is a diagonal matrix of weights for SNP variances (initially **D** = **I**); **M** is the number of SNPs; and *p_i_* is the minor allele frequency of the *i*th SNP. Variance components, heritability (*h*^2^), and repeatability (*re*) were estimated using the average information-restricted maximum likelihood (AI-REML) procedure implemented in the AIREMLF90 (v 1.148) software from the BLUPF90 (v 1.70) programs [27].

For Model (1),

$$h^{2}=\frac{\sigma_{a}^{2}}{\sigma_{a}^{2}+\sigma_{e}^{2}}$$

For Model (2),

$$h^{2}=\frac{\sigma_{a}^{2}}{\sigma_{a}^{2}+\sigma_{pe}^{2}+\sigma_{e}^{2}}$$

$$re=\frac{\sigma_{a}^{2}+\sigma_{pe}^{2}}{\sigma_{a}^{2}+\sigma_{pe}^{2}+\sigma_{e}^{2}}$$

### 2.5. Weighted Single-Step Genome-Wide Association Study (WssGWAS)

Although male genotypes were incorporated during the imputation phase to increase haplotype diversity and improve phasing accuracy, only females with both genotype and phenotype data were retained for the ssGBLUP and WssGWAS analyses. Consequently, all analyzed individuals were diploid for the X chromosome, which was treated identically to the autosomes in the genomic relationship matrix. The WssGWAS model followed the genetic model by postGSF90 (v 1.78) software in the BLUPF90 (v 1.70) programs. The estimates of SNP effects and weights for WssGWAS analyses (five iterations) were calculated using an iterative method as described by Wang, Misztal, Aguilar, Legarra and Muir [11]. The iteration increased the weights of SNPs with large effects and decreased those with small effects. The iteration procedure can be described as follows:

Step 1: Initialization, let *t* = 1, $D_{\left(t\right)}=I$, $G_{\left(t\right)}=\lambda ZD_{\left(t\right)}Z$, and $\lambda =1/\sum_{i}^{m}2p_{i}\left(1-p_{i}\right)$;
Step 2: GEBV estimation, calculate GEBVs $\left(\hat{\alpha }\right)$ via ssGBLUP with $H^{-1}=A^{-1}+\left[\begin{array}{cc} 0 & 0\\ 0 & G^{-1}-A_{22}^{-1} \end{array}\right]$;
Step 3: Marker effects calculation, obtain marker effects via ${\hat{g}}_{\left(t\right)}=\lambda D_{\left(t\right)}Z^{\prime }G_{\left(t\right)}^{-1}\hat{\alpha }$;
Step 4: SNP weights calculation, get SNP weights for the next iteration via $d_{i\left(t+1\right)}={\hat{g}}_{i\left(t\right)}^{2}p_{i}\left(1-p_{i}\right)$;
Step 5: SNP weights normalization. rescale the weights to keep the total genetic variance constant via $D_{\left(t+1\right)}=\frac{tr\left(D_{\left(1\right)}\right)}{tr\left(D_{\left(t+1\right)}\right)}D_{\left(t+1\right)}$;
Step 6: Weighted **G** construction, calculate **G** for the next iteration via $G_{\left(t+1\right)}=\lambda ZD_{\left(t+1\right)}Z$;
Step 7: Let *t* = *t* + 1 and loop to step 2 [8].

The percentage of the total additive genetic variance explained by the *i*th region was calculated as follows:

$$\frac{Var\left(a_{i}\right)}{\sigma_{a}^{2}}\times 100\%=\frac{Var(\sum_{j=1}^{20}Z_{j}{\hat{\mu }}_{j})}{\sigma_{a}^{2}}\times 100\%$$

where *a_i_* is the genetic value of the *i*th region that consists of contiguous 20 SNPs, $\sigma_{a}^{2}$ is the total additive genetic variance, ***Z**_j_* is a vector of gene content of the *j*th SNP for all individuals, and ${\hat{\mu }}_{j}$ is the marker effect of the *j*th SNP within the *i*th region [27]. Significant genomic regions were defined as windows of 20 adjacent SNPs explaining ≥0.2% of the total additive genetic variance.

### 2.6. Identification of Candidate Genes and Function Enrichment Analysis

Genomic windows of 20 consecutive SNPs that explained 0.2% or more of the total additive genetic variance, based on the WssGWAS analyses, were considered to be associated with the studied traits. A Manhattan plot was created using the ‘ggplot2’ (v 3.5.1) package [28] in R-4.3.3. Genes were annotated on the basis of the starting and ending coordinates of each window (using the ARS-UCD1.2 assembly as the reference genome; GCA_002263795.2) using the ‘Biomart’ (v 2.62.0) package [29] in R-4.3.3. Kyoto Encyclopedia of Genes and Genomes (KEGG) pathways and Gene Ontology (GO) terms were enriched via the ‘clusterProfiler’ (v 4.6.2) package [30] in R-4.3.3. All genes annotated within the significant QTL windows were used as the background gene list. Enrichment was assessed against the full set of protein-coding genes in the Bos taurus genome. The FDR method was applied to correct for multiple testing, with a significance threshold of adjusted *p* < 0.05. Candidate genes were prioritized based on a combination of two criteria: (1) Location within a QTL window explaining a relatively high proportion of genetic variance (top windows per trait) and (2) established biological relevance to reproductive processes as reported in the published literature and functional databases. The complete list of annotated genes and enriched GO terms for all traits is provided in Supplementary Table S4.

## 3. Results

### 3.1. Descriptive Statistics

Descriptive statistics for the eight fertility traits are summarized in Table 1. The number of animals with phenotypic records ranged from 115,517 (CI) to 161,172 (ICF), while those with genotypes ranged from 9943 to 13,484. Across all traits, coefficients of variation ranged from 10.77% (AFC) to 206.27% (IFL_H), indicating substantial phenotypic variability, particularly for interval traits. Detailed means, standard deviations, and record counts for each individual trait are provided in the table.

### 3.2. Heritability and Repeatability Estimates

**Figure 2:**
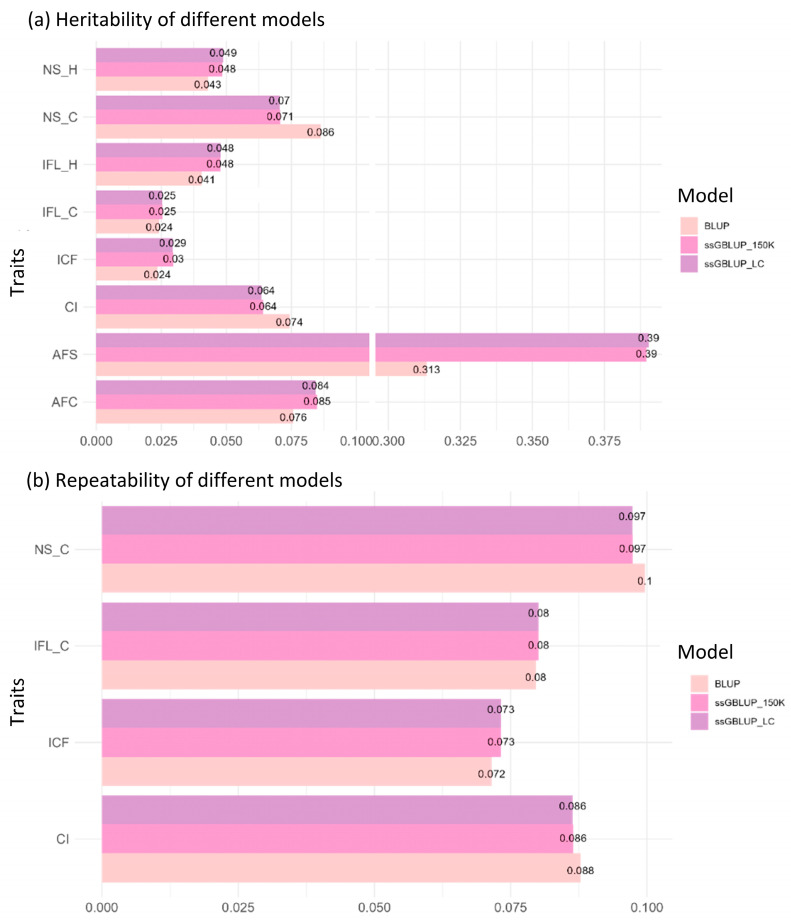
Heritability and repeatability estimation based on different models and different chips. (**a**) Heritability estimates of eight fertility traits derived from three models: Pedigree-based BLUP, single-step genomic BLUP with 150K SNP chip (ssGBLUP_150K), and single-step genomic BLUP with liquid-phase SNP chip (ssGBLUP_LC). (**b**) Repeatability estimates of four fertility traits with repeated records (NS_C, IFL_C, ICF, CI) using the same three models. AFS: Age at first service in heifer; AFC: Age at first calving in heifer; NS_H: Number of services for heifers; IFL_H: Interval from first to last inseminations in heifer; ICF: Interval from calving to first service; CI: Calving interval in cow; NS_C: Number of services for cows; and IFL_C: Interval from first to last inseminations in cows.

Figure 2 shows the heritability and repeatability computed based on distinct models (PBLUP and ssGBLUP) as well as different chips (150 K and liquid chip). Based on the ssGBLUP_150K model, the heritability estimates ranged from 0.025 (IFL_C) to 0.390 (AFS). Among all fertility traits, AFS had moderate heritability, while the other seven traits had low heritability. For the ssGBLUP_LC model, the heritability estimates for all fertility traits were similar to those from the ssGBLUP_150K model, ranging from 0.025 ± 0.002 (IFL_C) to 0.390 ± 0.008 (AFS). Notably, the integration of genomic information (ssGBLUP) generally enhanced the heritability estimates when compared with PBLUP, except for NS_C. For the cow fertility traits, the repeatability estimated by the ssGBLUP_LC and ssGBLUP_150K models are the same, which were 0.097 ± 0.003 (NS_C), 0.080 ± 0.003 (IFL_C), 0.073 ± 0.002 (ICF), and 0.086 ± 0.003 (CI), respectively. Compared with ssGBLUP, the repeatability estimates of NS_C (0.100 ± 0.003) and CI (0.088 ± 0.004) by the BLUP model is higher, while the repeatability estimate of ICF (0.072 ± 0.002) by the BLUP model is lower.

### 3.3. WssGWAS Results of Fertility Traits

**Figure 3:**
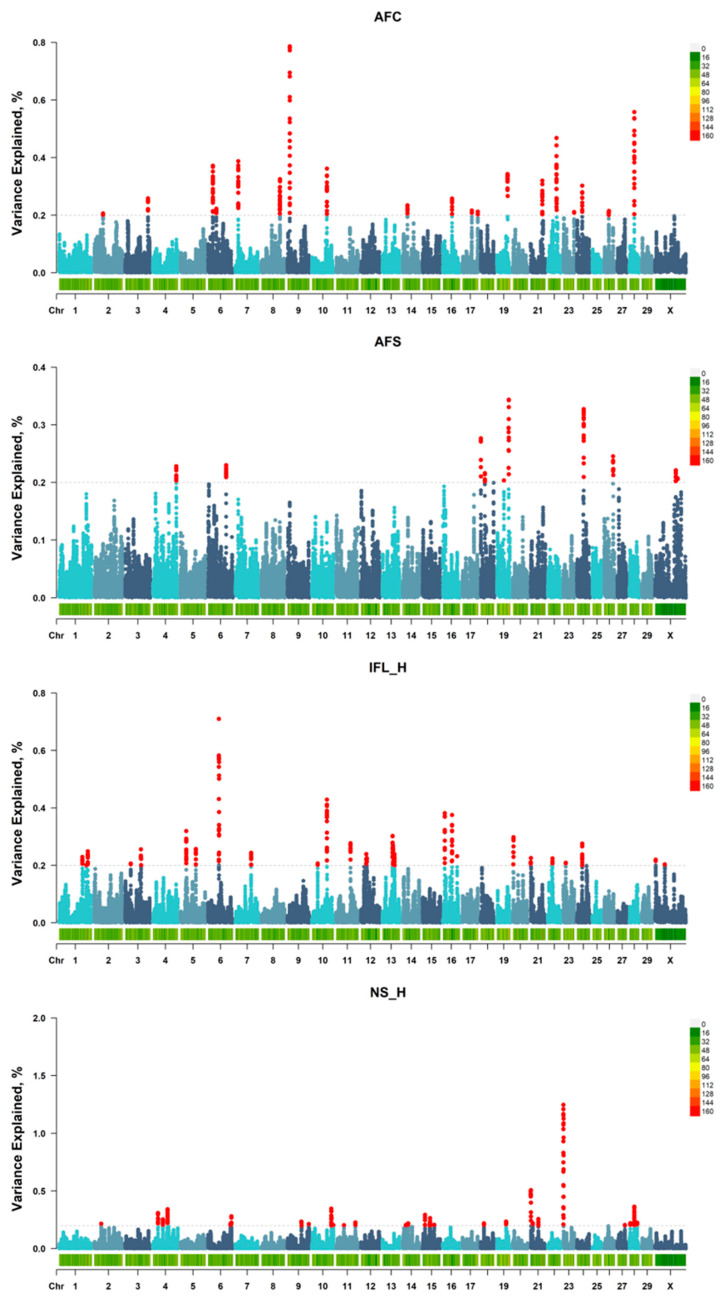

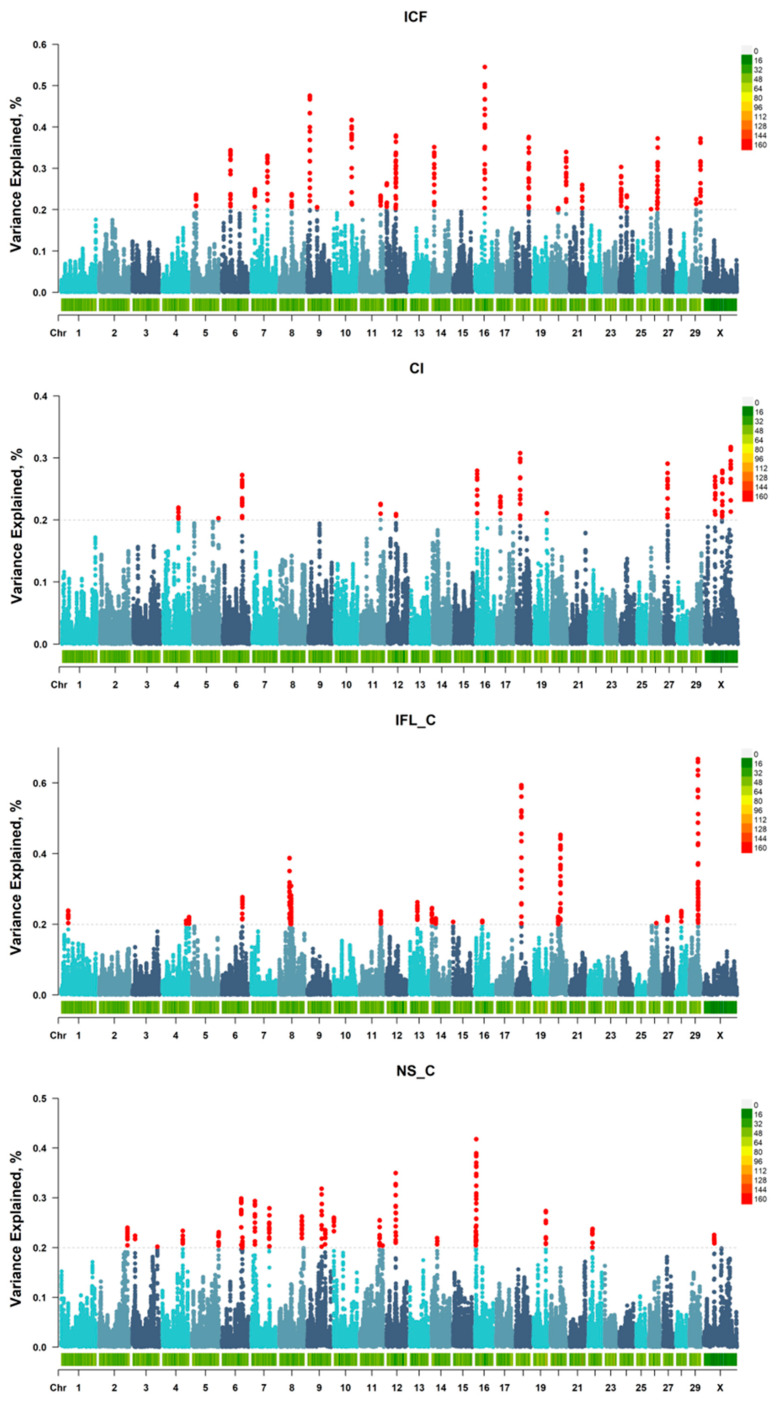
Manhattan plot illustrating the proportion of genetic variation in eight fertility traits explained by a window of 20 adjacent SNPs in Holstein cattle. AFS: Age at first service in heifer; AFC: Age at first calving in heifer; NS_H: Number of services for heifers; IFL_H: Interval from first to last inseminations in heifer; ICF: Interval from calving to first service; CI: Calving interval in cow; NS_C: Number of services for cows; and IFL_C: Interval from first to last inseminations in cows. Red dots represent genomic windows exceeding the 0.20% threshold of total additive genetic variance. Blue points represent other genomic windows.

Figure 3 presents the genetic variance of each genomic window after performing WssGWAS with five iterations. A total of 162 QTL windows were identified, each explaining more than 0.2% of the total genetic variation. Supplementary Figure S1 shows the overlap of significant SNP windows associated with fertility traits in Chinese Holstein cattle.

#### 3.3.1. Heifer Fertility Traits

The WssGWAS effectively accounted for the genetic variation within 20 SNP genomic windows for each heifer fertility trait. As shown in Figure 2 and Supplementary Table S2, a total of 9, 19, 27 and 20 genomic regions reached the predefined threshold (0.2% of the total genetic variance) for AFS (2.12%), AFC (5.15%), NS_H (7.91%) and IFL_H (7.37%), respectively. Most of the significant windows were located in the *Bos taurus autosome* (BTA) BTA4, BTA6, BTA10, BTA16, BTA19, BTA21, and BTA28. Specifically, the QTL regions were distributed across 7 (AFS), 16 (AFC), 16 (NS_H), and 14 (IFL_H) chromosomes. Among all identified QTL regions, the QTL region on BTA23: 0.02–1.35 Mb explained the largest genetic variance (1.245%) for NS_H.

Pleiotropic effects were observed through the shared QTL regions between different traits. Three shared QTL regions were found between AFC and IFL_H (BTA10: 72.28–73.16 Mb, BTA16: 43.74–44.27 Mb, and BTA24: 25.73–25.98 Mb), which encompassed a total of 29 common candidate genes. AFC and NS_H shared a QTL region on BTA28: 22.94–23.53 Mb, containing one common candidate gene (*LRRTM3*). Furthermore, there was a shared QTL region located in BTA21: 0.36–2.69 Mb between NS_H and IFL_H, which contained six common candidate genes. The top three most significant QTL regions for each heifer fertility trait are shown in Table 3. These windows individually explained between 0.249% and 1.245% of the genetic variance, with the cumulative explained genetic variance for the top three windows being 0.826%, 1.233%, 2.112%, and 1.394% for AFS, AFC, NS_H, and IFL_H, respectively. Among these regions, the key candidate genes were identified, including 14 for AFS, two (*LRRTM3*, *FHIT*) for AFC, eight (*KHDRBS2*, *UBE3A*, *NDN*, *MAGEL2*, *SNORD116*, *SNORD115*, *SNORD109A*, *LRRTM3*) for NS_H, and 14 for IFL_H. These are shown in Table 3.

#### 3.3.2. Cow Fertility Traits

The WssGWAS identified a total of 79 QTL regions across the four cow fertility traits, with detailed results presented in Figure 2, Table 4, and Supplementary Table S3. The number of QTL regions detected per trait ranged from 13 for CI to 24 for ICF, and the proportion of genetic variance explained by individual windows varied from 0.201% to 0.667%. The top three windows per trait cumulatively explained between 0.914% (CI) and 1.713% (IFL_C) of the genetic variance (Table 4), with IFL_C showing the most prominent signals, consistent with its relatively higher phenotypic variability.

A total of nine overlapping QTL windows were identified across traits, suggesting potential pleiotropic effects. Of particular interest, two shared regions were detected between ICF and NS_C (on BTA7 and BTA12), and one region on BTA20 (34.34–35.02 Mb) overlapped between ICF and IFL_C, harboring *DAB2*, a gene previously implicated in female reproductive function. Five shared regions were found between CI and NS_C, distributed across BTA6, BTA11, BTA16, BTA19, and BTAX, collectively encompassing 19 common candidate genes. Additionally, one overlapping window on BTA6 (91.84–92.51 Mb) was shared between NS_C and IFL_C and contained seven candidate genes.

Annotation of the top three windows per trait revealed several biologically relevant candidate genes. Among these, *ITFG1* was associated with CI, while *CR2*, *PCDH9*, *SYNCRIP*, *SNX14*, *NT5E*, and *SNORD50* were linked to NS_C. A total of 28 and 33 genes were annotated in the top windows for ICF and IFL_C, respectively, with several showing established roles in ovarian function, uterine receptivity, or early embryonic development. The full list of QTL regions and annotated genes across all windows is provided in Supplementary Table S3.

### 3.4. Functional Enrichment Analyses and Key Candidate Genes

A total of 645 candidate genes were annotated for all fertility traits according to the Ensembl database. Table 5 shows some candidate genes which are the most closely associated with fertility traits, including *ovulation-inducing factor 2* (*OVOS2*); *prostate transmembrane protein, androgen-induced 1* (*PMEPA1*); *testis, prostate and placenta-expressed protein* (*TEPP*); the *RAG* family (*pregnancy-associated glycoproteins*) and so on.

**Figure 4:**
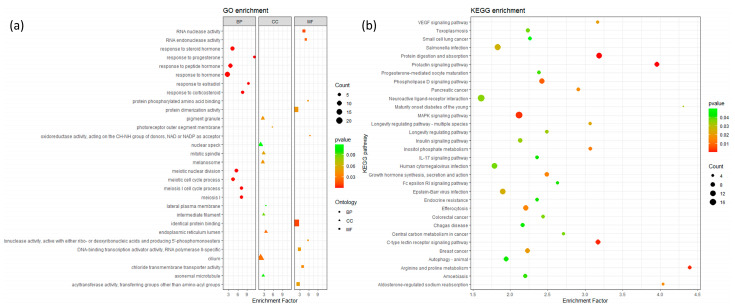
Functional annotation diagram of important candidate genes. (**a**) Dot plot with color gradient drawn for 20 items involved in the important candidate genes in the enrichment analysis GO items, where the size of the circle represents the number of genes included in the GO items, and the larger the number of involved genes, the larger the circle (BP, biological process; CC, cellular component; MF, molecular function). (**b**) Dot plot of KEGG analysis results. Bubble diagram of all KEGG pathways with FDR-corrected *p* < 0.05, where the size of the circle represents the number of genes included in the pathway, and the larger the number of involved genes, the larger the circle.

Figure 4 presents the results of GO enrichment and KEGG pathway analyses for the 645 candidate genes associated with fertility traits. From the GO enrichment analysis, reproduction-related hormones (such as progesterone, steroids, estradiol, corticosteroids, etc.) were derived; from the KEGG pathway analysis, pathways including progesterone-mediated oocyte maturation, prolactin signaling pathway, and the longevity—regulating signaling pathway were identified.

## 4. Discussion

In this study, we conducted an extensive genetic analysis of eight fertility traits within a substantial Chinese Holstein population, utilizing phenotypic records from over 160,000 cows alongside genotypic data obtained from both liquid-phase and 150 K chips. Genetic parameters were estimated employing PBLUP and ssGBLUP models, and genomic regions as well as candidate genes associated with fertility traits were identified through WssGWAS. The results directly address our research objectives. Specifically, the heritability estimates confirmed that most fertility traits are lowly heritable, whereas age at first service exhibited moderate heritability (~0.39), delineating traits amenable to direct selection from those requiring indirect approaches. Furthermore, the WssGWAS identified a total of 162 QTL intervals and highlighted promising candidate genes such as *RARRES2*, *TEPP*, *HSPA8*, and the *RAG* family, which are biologically implicated in reproductive processes. These findings provide concrete genomic targets for future validation and selection strategy refinement. To the best of our knowledge, this research represents one of the pioneering efforts to integrate GBTS-based liquid chips with WssGWAS for the genetic dissection of fertility traits in Chinese Holstein cattle, thereby offering valuable insights into the genetic architecture of these traits with low heritability.

### 4.1. Descriptive Statistics of Fertility Traits

In this study, a systematic descriptive statistical analysis was performed on the fertility traits of Chinese Holstein cattle. When compared with the findings of our previous research [31,32], which examined the same population from 2014 to 2018, there has been a significant reduction in the interval from calving to first service (ICF), decreasing from 82.1 days to 67.8 days. Additionally, the interval from first to last insemination in heifers (IFL_H) has been reduced from 32.1 days to 28.2 days, and the interval from first to last insemination in cows (IFL_C) has decreased markedly from 79.5 days to 58.9 days. Collectively, these reductions in ICF, IFL_H, and IFL_C strongly indicate an improvement in the management practices at the ranch. This improvement may be attributed to various factors, including enhanced breeding strategies, improved animal care, or more efficient reproductive management practices implemented at the ranch. In comparison to the Holstein cattle population in southern China [33], it is apparent that the age at first service (AFS) in heifers, age at first calving (AFC) in heifers, and calving interval (CI) in cows are all lower in the Beijing population. This regional disparity can be attributed to two primary factors: firstly, the large-scale, standardized management practices employed by dairy farms in Beijing, which minimize environmental variability in reproductive performance; and secondly, the pronounced impact of heat stress on dairy cattle in southern China, which adversely affects endocrine function, estrous cyclicity, and endometrial receptivity, thereby extending intervals and diminishing reproductive efficiency [34,35,36]. These findings underscore the necessity of accounting for environmental influences and genotype-by-environment interactions in the genetic evaluation of dairy cattle fertility traits within China.

### 4.2. Genetic Parameter Estimation

The heritability estimates for fertility traits in this study were low (except AFS), ranging from 0.024 ± 0.002 (IFL_C and ICF) to 0.085 ± 0.006 (AFC), with most traits showing heritability below 0.1. These results are highly consistent with the global consensus that dairy cattle fertility traits are typically low-heritability traits [37,38,39]. The moderate heritability of AFS (0.390 ± 0.008) observed here is also consistent with previous reports, as age at first service is a trait that is less affected by random environmental factors and has a more stable genetic basis [40].

Notably, we found that ssGBLUP models (integrating genomic information) yielded higher heritability estimates for nearly all traits when compared with traditional PBLUP models, which is consistent with the well-documented advantage of single-step methods in capturing additive genetic variance more accurately [25,41]. This improvement is particularly valuable for low-heritability fertility traits, as it reduces the bias in genetic parameter estimation caused by incomplete pedigree information and limited genotyped samples [12]. When comparing the two chip types, the heritability estimates from the liquid chip were nearly identical to those from the 150 K chip, with no statistically significant differences observed. This may be due to the fact that the remaining chip loci of the two chips after quality control are similar, and the InDels of the liquid-phase chips were not retained during the imputation.

For the cow fertility traits (ICF, CI, NS_C, IFL_C), the repeatability estimates ranged from 0.072 ± 0.002 (ICF) to 0.100 ± 0.003 (NS_C), which are consistent with previous reports in Holstein cattle [31,33,38]. For CI, the heritability of this study (0.074 ± 0.003) was slightly higher than that in the Jersey cattle population (0.05 ± 0.05), while the repeatability (0.088 ± 0.004) was slightly lower than that in the Jersey cattle population (0.15 ± 0.03) [42], which was mainly caused by breed differences, and the sample size of our study population (approximately 110,000 cows) was much larger than that of the Jersey cattle population (1201 cows).

### 4.3. QTL and Candidate Genes Identified via WssGWAS

Using WssGWAS, we identified a total of 162 QTL windows (explaining ≥0.2% of the additive genetic variance) associated with the eight fertility traits, distributed across multiple autosomes. The WssGWAS method proved to be effective for the genetic dissection of these low-heritability traits, as it integrated pedigree, phenotypic, and genomic data to improve the power of QTL detection, which is consistent with previous studies [8,11].

Among the candidate genes annotated in the significant QTL windows, several have well-documented roles in reproductive physiology, providing strong biological support for our findings. For example, *ovostatin 2* (*OVOS2*), identified for ICF, encodes a protein involved in folliculogenesis and ovulation regulation, and has been previously associated with reproductive performance in cattle of South Africa [43]. *Testis-specific kinase 1* (*TESK1*), also identified for ICF, plays a critical role in spermatogenesis and early embryonic development, and has been reported as a candidate gene for goat male fertility [44] in previous studies. *Heat shock 70 kDa protein 8* (*HSPA8*), identified for NS_H, is a member of the heat shock protein family, which is involved in endometrial receptivity and embryo implantation, and has been linked to dairy cattle fertility traits in previous research [45,46]. Additionally, we identified several genes with known roles in reproductive processes, including *TEPP* (for IFL_C), a conserved gene specifically expressed in reproductive tissues including the testis, prostate, and placenta; *RARRES2* (for AFS), which is possibly involved in the *RARRES2/CMKLR1* system in metabolic and reproductive parameters in Holstein dairy cows [47]; *PMEPA1* (for IFL_H), a transmembrane protein that negatively regulates androgen receptor and TGF-β signaling pathways, and which is associated with the interval from first to last insemination in dairy cattle in its modulation of reproductive hormone secretion, uterine environment, and embryonic survival, and with its genetic variants and expression levels directly influencing breeding efficiency and the number of inseminations required for conception [48]; *TXNDC8* (for NS_C), whose content in sperm reflects sperm chromatin structure, pregnancy establishment, and incidence of multiple births after assisted reproductive therapy (ART) [49]; and *PAG* family genes (for IFL_C), which encode pregnancy-associated glycoproteins that are critical for pregnancy maintenance and fetal development in ruminants [50,51]. We note that certain GO terms related to male fertility (e.g., spermatogenesis, sperm motility) were enriched among the candidate genes. While our study focuses on female phenotypes, many genes governing core reproductive processes—such as meiosis, cell cycle regulation, and gamete development—exhibit functional pleiotropy and are expressed in both male and female reproductive tissues. The female conception rate is intrinsically linked to male fertility, as fertilization success depends on both the oocyte and the spermatozoon. Conception outcomes in the field are inevitably influenced by the semen quality of the service sires. To account for this, our statistical models included sire- and insemination-related effects as fixed effects, meaning that the genetic signals captured for female fertility traits may partially reflect the biological interplay between the maternal uterine environment and the paternal gamete. This is a recognized phenomenon in fertility GWAS where traits measured on one sex can reveal genetic variants acting through the other sex. Therefore, the enrichment of these terms likely reflects the fundamental roles of these genes in reproduction, rather than a male-specific bias.

Notably, we detected overlapping QTL regions across multiple traits, including shared regions between AFC and IFL_H, NS_H and IFL_H, and ICF and NS_C. These overlapping regions suggest the presence of pleiotropic QTLs that influence multiple fertility traits, which is consistent with the strong genetic correlations reported between fertility traits in dairy cattle [31,52]. These pleiotropic loci are particularly valuable for genomic breeding, as they can be used to simultaneously improve multiple fertility traits in selection programs.

Some of the QTL regions and candidate genes identified in this study have not been previously reported in dairy cattle fertility GWAS, representing novel findings that expand our understanding of the genetic architecture of fertility traits in Chinese Holstein cattle. The differences between our results and previous studies may be attributed to the population-specific genetic background and the use of the wssGWAS method (which has a higher sensitivity for small-effect loci).

### 4.4. The Strengths and Limitation of WssGWAS

WssGWAS can optimize effect estimation in key genomic regions through weighted models and integrate functional annotations (e.g., gene pathways, RNA-seq data) to improve the biological interpretation of results [53]. Despite the promising application prospects of WssGWAS and liquid chip technology, several challenges remain in the genetic analysis of Holstein fertility traits. First, data quality control: accurate recording of fertility trait phenotypes (e.g., age at first breeding, pregnancy rate) is essential. Weller et al. [40] has indicated that early phenotypes (such as age at first breeding) have higher heritability and are more suitable for GWAS, while the high genotyping accuracy of liquid chips can reduce false positives caused by genotype errors [54]. Second, cross-population validation and functional mechanism verification: Höglund et al. [55] has validated QTL regions through northern European and Chinese Holstein populations and found multiple shared loci (such as BTA13 and BTA28). WssGWAS combined with cross-population meta-analysis can enhance the reliability of results [56] and candidate genes can be validated through functional experiments (e.g., gene editing) [57]. In the future, combining multi-omics data (e.g., epigenome, metabolome) and cross-species functional annotations will be crucial for comprehensively understanding the molecular mechanisms of fertility traits [53].

However, SNP weight calculation relies on iterative convergence. If the initial model has a large deviation, it may lead to unstable results [58,59]. Different window definitions (such as 10 to 50 SNPS) may affect the division of significant regions and need to be adjusted in combination with the biological background [12,60,61]. Although it theoretically supports multi-trait models, the computational complexity is high, and reliability may be reduced when there is low correlation between traits [62]. In addition, existing methods rarely systematically integrate prior information such as eQTL and epigenetics. In the future, weight distribution can be optimized by combining functional annotations [9,10].

### 4.5. The Limitation of the Present Study

Several limitations of the present study should be acknowledged. Initially, while we incorporated genotypes from 150 K and liquid-phase chips with high imputation accuracy, our comparative analysis of these chips was exploratory in nature rather than statistically conclusive. The study was not initially designed to formally establish equivalence between the platforms. Despite the Venn diagram and imputation metrics indicating that the 150 K and liquid-phase chips offer comparable marker coverage post-harmonization, a formal cross-validation assessing the prediction accuracies of genomic estimated breeding values (GEBVs) between the two platforms was not conducted. Such an analysis would further substantiate the interchangeability of these platforms in routine genetic evaluations. Second, the MAF ≥ 0.05 filter, while necessary to ensure robust SNP effect estimation at our sample size, may have excluded rare variants with potentially large effects. Third, the exclusion of InDels during genotype harmonization, although methodologically justified, means that certain structural variants contributing to fertility may have been missed. Fourth, right-censored data from cows culled early due to reproductive failure could introduce selection bias, potentially leading to the underestimation of genetic variance for infertility traits. Fifth, the findings are specific to the Chinese Holstein population under the management conditions studied, and their transferability to other populations warrants further investigation. Despite these caveats, the core QTL regions and candidate genes identified with conservative criteria remained stable across sensitivity analyses, supporting the reliability of our conclusions.

## 5. Conclusions

Our study provides a comprehensive genetic dissection of eight fertility traits in the Chinese Holstein population using WssGWAS. The heritability estimates confirmed that most fertility traits are lowly heritable (0.024–0.085), whereas age at first service showed moderate heritability (0.390), highlighting its potential for direct selection. Our study revealed 645 candidate genes associated with eight fertility traits in the Chinese Holstein population using WssGWAS. Based on previous research into related fertility traits and the biological functions of these genes, several further promising candidate genes for fertility traits are proposed, including *RARRES2*, *TEKT2*, *OVOS2*, *HSPA8*, *TEPP* and the *RAG* family. These findings lay a solid foundation for research into the genetic mechanism of fertility traits and provide information for the marker-assisted selection or genome selection for fertility traits in Holstein. In the future, it is necessary to further combine multi-omics data (e.g., epigenome, metabolome) and cross-species functional annotation to comprehensively understand the molecular mechanisms of fertility traits.

## Figures and Tables

**Table 1:** Descriptive statistics of eight fertility traits in this study.

Traits ^a^	N_cow ^b^	N_rec ^c^	N_gen ^d^	Mean ± SD	Min	Max	CV ^e^ (%)
**AFS**	154,113	154,113	11,280	463.90 ± 70.83	270	900	15.27
**AFC**	130,332	130,332	11,022	765.12 ± 82.39	500	1100	10.77
**NS_H**	134,141	134,141	10,906	1.67 ± 1.19	1	10	71.26
**IFL_H**	159,876	159,876	12,038	28.21 ± 58.19	0	355	206.27
**ICF**	161,172	345,751	13,096	67.78 ± 21.65	20	230	31.94
**CI**	115,517	238,277	9943	395.02 ± 64.76	280	600	16.39
**NS_C**	120,394	245,501	11,087	2.40 ± 1.80	1	10	75.00
**IFL_C**	146,965	317,565	13,484	58.88 ± 77.00	0	355	130.77

^a^ AFS: Age at first service in heifer; AFC: Age at first calving in heifer; NS_H: Number of services for heifers; IFL_H: Interval from first to last inseminations in heifer; ICF: Interval from calving to first service; CI: Calving interval in cow; NS_C: Number of services for cows; IFL_C: Interval from first to last inseminations in cows. ^b^ N_cow: Numbers of cows with phenotypic records. ^c^ N_rec: Numbers of records. ^d^ N_gen: Numbers of animals with genotypic data. ^e^ CV: Coefficient of variation.

**Table 2:** Fixed effects included in the models for genetic parameter estimation of all fertility traits.

Traits ^a^	Fixed Effects ^b^									
	HYB	HYC	HYI	YMB	YMC	YMI	ST	SC	MF	P
**AFS**	√			√			√			
**AFC**	√			√			√	√		
**NS_H**			√			√	√		√	
**IFL_H**			√			√	√	√	√	
**ICF**		√			√		√		√	√
**CI**		√			√		√		√	√
**NS_C**			√			√	√		√	√
**IFL_C**			√			√	√	√	√	√

^a^ AFS: Age at first service in heifer; AFC: Age at first calving in heifer; NS_H: Number of services for heifers; IFL_H: Interval from first to last inseminations in heifer; ICF: Interval from calving to first service; CI: Calving interval in cow; NS_C: Number of services for cows; and IFL_C: Interval from first to last inseminations in cows. ^b^ HYB: The fixed effect of herd-birth year; HYC: The fixed effect of herd-calving year; HYI: The fixed effect of herd-year of the first service in this lactation; YMB: The fixed effect of birth year-birth month; YMC: The fixed effect of calving year-calving month; YMI: The fixed effect of year-month of first service in this lactation; ST: The fixed effect of service technician; SC: The fixed effect of semen type (conventional semen = 1; sexed semen = 2); MF: The fixed effect of age at first service in heifer (5 levels, included 270 d–439 d, 440 d–469 d, 470 d–499 d, 500 d–529 d, 530 d–900 d); and P: The fixed effect of parity.

**Table 3:** The top three most significant QTL regions for heifer fertility traits.

Trait ^a^	Chr ^b^	Regions (Mb)	Number of SNPs	gVar (%) ^c^	Number of Genes	Genes
**AFS**	BTA24	32.96–33.71	37	0.292	8	*LAMA3*, *U6*, *TTC39C*, *ANKRD29*, *NPC1*, *RIOK3*, *TMEM241*, *CABLES1*
	BTA19	56.56–56.68	32	0.285	6	*SMIM6*, *SMIM5*, *LLGL2*, *TSEN54*, *RECQL5*, *CASKIN2*
	BTA18	0.49–0.93	25	0.249	0	*/*
**AFC**	BTA9	10.93–11.26	42	0.504	0	*/*
	BTA28	22.91–23.57	38	0.400	1	LRRTM3
	BTA22	41.19–42.03	43	0.329	1	*FHIT*
**NS_H**	BTA23	0.02–1.35	45	1.245	1	*KHDRBS2*
	BTA21	0.36–2.77	33	0.506	6	*UBE3A*, *NDN*, *MAGEL2*, *SNORD116*, *SNORD115*, *SNORD109A*
	BTA28	22.94–23.53	33	0.361	1	*LRRTM3*
**IFL_H**	BTA6	51.96–53.03	42	0.583	1	*PCDH7*
	BTA10	72.28–73.83	60	0.429	13	*COX7C*, *LRRC9*, *PCNXL4*, *DHRS7*, *PPM1A*, *SIX6*, *SIX1*, *SIX4*, *MNAT1*, *TRMT5*, *SLC38A6*, *PRKCH*, *TMEM30B*
	BTA16	8.3–9.05	33	0.382	0	*/*

^a^ AFS: Age at first service in heifer; AFC: Age at first calving in heifer; NS_H: Number of services for heifers; IFL_H: Interval from first to last inseminations in heifer. ^b^ Chr: Chromosome. ^c^ gVar (%): Genetic variance explained by a window consisting of 20 adjacent SNPs.

**Table 4:** The top three most significant QTL regions for cow fertility traits.

Trait ^a^	Chr ^b^	Regions (Mb)	Number of SNPs	gVar (%) ^c^	Number of Genes	Genes
**ICF**	BTA16	43.17–45.05	39	0.545	24	*CASZ1*, *PEX14*, *DFFA*, *CORT*, *APITD1*, *PGD*, *KIF1B*, *SNORD77*, *EXOSC10*, *SRM*, *MASP2*, *TARDBP*, *UBIAD1*, *MTOR*, *ANGPTL7*, *UBE4B*, *RBP7*, *NMNAT1*, *CTNNBIP1*, *CLSTN1*, *PIK3CD*, *TMEM201*, *SLC25A33*, *SPSB1*
	BTA9	10.94–11.21	38	0.476	0	*/*
	BTA10	80.55–81.21	36	0.417	4	*ZFP36L1*, *ACTN1*, *RAD51B*, *DCAF5*
**CI**	BTAX	117.13–118.54	34	0.315	0	*/*
	BTA18	15.63–15.93	34	0.308	1	*ITFG1*
	BTA27	15.68–16.15	30	0.291	0	*/*
**NS_C**	BTA16	5.13–5.98	39	0.418	1	*CR2*
	BTA12	40.48–41.87	35	0.350	1	*PCDH9*
	BTA9	64.13–65.25	29	0.318	4	*SYNCRIP*, *SNX14*, *NT5E*, *SNORD50*
**IFL_C**	BTA29	38.54–39.76	61	0.667	10	*PAG-15*, *PAG4*, *PAG7*, *PAG20*, *PAG-21*, *PAG1*, *PAG19*, *PAG17*, *MGC157405*, *MGC157408*
	BTA18	25.02–25.98	39	0.593	23	*HERPUD1*, *NLRC5*, *CPNE2*, *NIP30*, *RSPRY1*, *ARL2BP*, *PLLP*, *CCL22*, *CX3CL1*, *CCL17*, *CIAPIN1*, *COQ9*, *POLR2C*, *DOK4*, *CCDC102A*, *ADGRG5*, *SLC12A3*, *ADGRG3*, *CCDC135*, *KATNB1*, *KIFC3*, *CNGB1*, *TEPP*
	BTA20	43.57–44.32	48	0.453	0	*/*

^a^ ICF: Interval from calving to first service; CI: Calving interval in cow; NS_C: Number of services for cows; and IFL_C: Interval from first to last inseminations in cows. ^b^ Chr: Chromosome. ^c^ gVar (%): Genetic variance explained by a window consisting of 20 adjacent SNPs.

**Table 5:** Partial candidate genes for all fertility traits in this study.

Gene	Description	Chromosome	Trait
***RARRES2***	Retinoic acid receptor responder (tazarotene induced) 2	4	AFS
***TEKT2***	Tektin 2 (testicular)	3	AFC
***OVOS2***	Follicle-stimulating hormone 2	5	ICF
***CABS1***	Calcium-binding protein, spermatid-specific 1	6	AFS
***TESK1***	Testis-specific kinase 1	8	ICF
***TXNDC8***	Thioredoxin domain containing 8 (spermatozoa)	8	NS_C
***LGB***	Progesterone-related endometrial protein	11	ICF
***STRBP***	Spermatid perinuclear RNA binding protein	11	IFL_C
***PMEPA1***	Prostate transmembrane protein, androgen induced 1	13	IFL_H
***SOX17***	sex determining region Y (SRY)-box 17	14	AFC, CI
***HSPA8***	Heat shock 70 kDa protein 8	15	NS_H
***TEPP***	Testis, prostate and placenta-expressed protein	18	IFL_C
***RAG***	Pregnancy-associated glycoprotein	29	IFL_C

## Data Availability

The data presented in this study are available on request. These data are not publicly available to preserve the data privacy of the commercial farm.

## Supplementary Materials

The following supporting information can be downloaded at https://www.mdpi.com/article/10.3390/ani16111622/s1. Table S1: Genetic parameters of eight fertility traits; Table S2: Information of 20-SNP windows explaining more than 0.20% of total additive genetic variance for heifers fertility traits in Chinese Holstein cattle; Table S3: Information of 20-SNP windows explaining more than 0.20% of total additive genetic variance for cows fertility traits in Chinese Holstein cattle; Table S4: Significant GO terms in candidate gene enrichment in WssGWAS for fertility traits in Chinese Holstein cattle. Figure S1: Venn diagrams showing the overlap of significant SNP windows associated with fertility traits in Chinese Holstein cattle. Supplementary_M&M.docx: S1. Phenotypic Data Processing and Trait Definition.

## Abbreviations

The following abbreviations are used in this manuscript:

Abbreviations	Full English name
AFC	Age at first calving in heifer
AFS	Age at first service in heifer
AI-REML	Average information-restricted maximum likelihood
CI	Calving interval in cow
GBTS	Genotyping by target sequencing
GO	Gene Ontology
ICF	Interval from calving to first service
IFL_C	Interval from first to last inseminations in cows
IFL_H	Interval from first to last inseminations in heifer
Indel	Insertion-deletion
KEGG	Kyoto Encyclopedia of Genes and Genomes
NS_C	Number of services for cows
NS_H	Number of services for heifer
WssGWAS	Weighted single-step genome-wide association studies
