# Supplementary material for: Genetic Parameters and Weighted Single-Step Genome-Wide Association Studies of Fertility Traits in Chinese Holstein
Source: Animals (Basel). 2026 May 26;16(11):1622. doi: 10.3390/ani16111622 (PMC13255800; doi:10.3390/ani16111622)
Supplement: Supplementary file 1 [file animals-16-01622-s001.zip › Supplementary_figure.pdf]

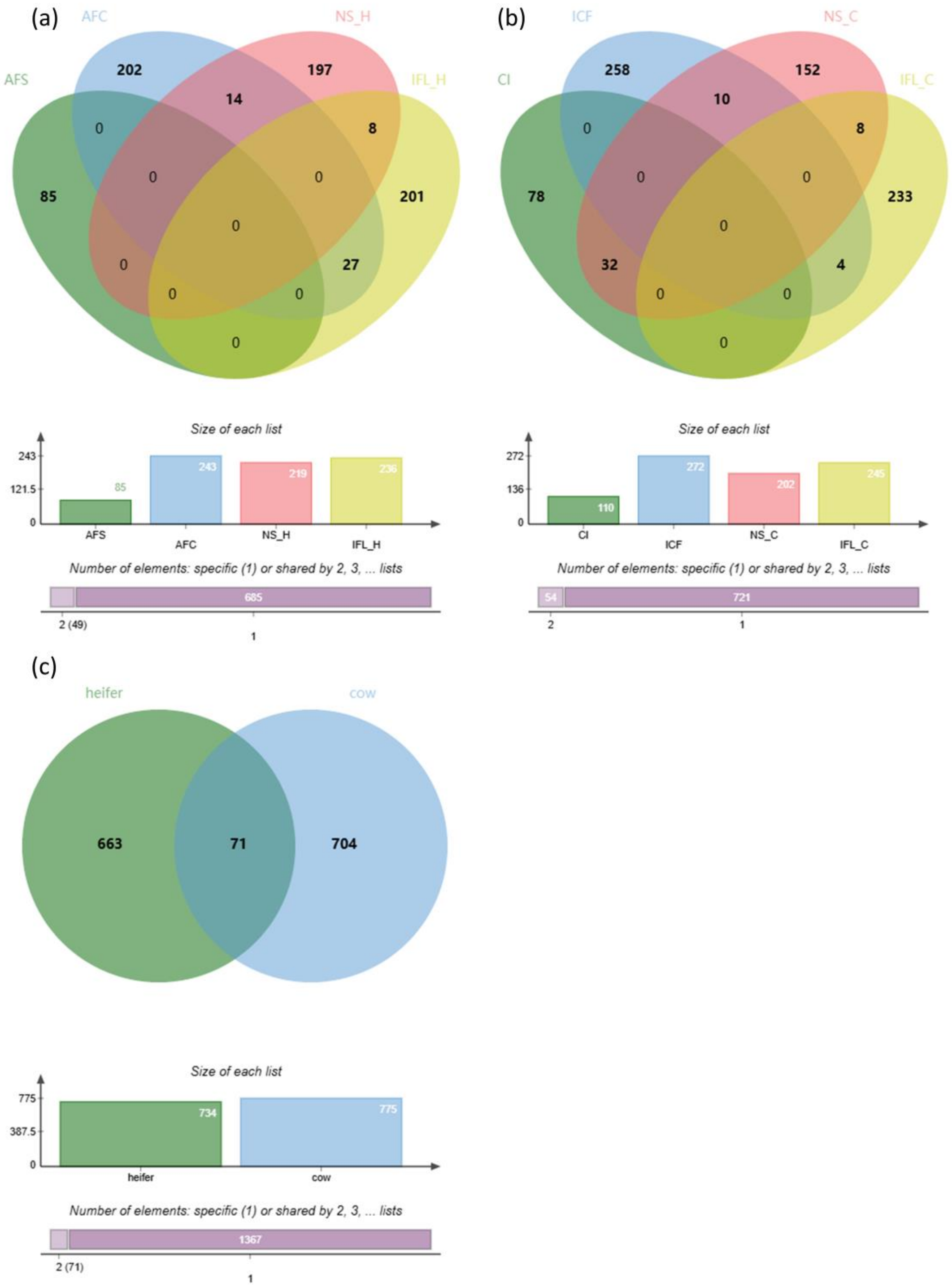


Figure S1. Venn diagrams showing the overlap of significant SNP windows associated with fertility traits in Chinese Holstein cattle. a) Overlap of significant SNP windows among four heifer fertility traits: age at first service (AFS), age at first calving (AFC), number of services for heifers (NS_H), and interval from first to last inseminations in heifers (IFL_H). b) Overlap of significant SNP windows among four cow fertility traits: calving interval (CI), interval from calving to first service (ICF), number of services for cows (NS_C), and interval from first to last

inseminations in cows (IFL_C). (c) Overlap of significant SNP windows between heifer and cow fertility trait sets. Numbers in the Venn diagrams represent the count of unique or shared significant SNP windows. The bar plots below each diagram show the total number of significant SNP windows for each trait or group.
