# Supplementary material for: Genetic Parameters and Weighted Single-Step Genome-Wide Association Studies of Fertility Traits in Chinese Holstein
Source: Animals (Basel). 2026 May 26;16(11):1622. doi: 10.3390/ani16111622 (PMC13255800; doi:10.3390/ani16111622)
Supplement: Supplementary file 1 [file animals-16-01622-s001.zip › Supplementary_M&M.pdf]

Supplemental Materials and Methods

S1. Phenotypic Data Processing and Trait Definition

S1.1 Event Record Types and Field Descriptions

Four types of event records were used for fertility trait calculation:

Calving events: All records with the "Event" field labeled as "Calving" were extracted. Each record contained 15 fields: Cow ID, Date, Event, Event Information, Parity, Days in Milk, Daily Milk Yield, Calving Ease, Birth Weight, Inseminator, HERD, FID, CE, SEX, and SB. Multiple records existed per cow, corresponding to different parities.

Insemination events: All records with the "Event" field labeled as "Insemination" were extracted. Each record contained 12 fields: Cow ID, Date, Event, Event Information, Parity, Days in Milk, Daily Milk Yield, Calving Ease, Birth Weight, Inseminator, HERD, and FID. Multiple records existed per cow per parity.

Birth events: All records with the "Event" field labeled as "Birth" were extracted. Each record contained 12 fields identical to those in insemination events. Only one record existed per cow.

Pregnancy Check events: All records with the "Event" field labeled as "Pregnancy Diagnosis" were extracted. Each record contained 12 fields identical to those in insemination events. Multiple records existed per cow per parity.

S1.2 Calculation Methods for fertility Traits

The eight fertility traits were calculated based on standardized event records. Herd, year and month of previous calving was included as a fixed effect in all subsequent genetic models.

Age at First Service (AFS): For heifers, the interval in days between the earliest insemination date and the birth date of the same individual.

Age at First Calving (AFC): For heifers, the interval in days between the earliest calving date and the birth date of the same individual.

Number of Services for Heifers (NS_H): Total number of inseminations between the first and last insemination of the first parity for heifers.

Interval from First to Last Inseminations in Heifer (IFL_H): Interval in days between the first and last insemination of the first parity for heifers.

Interval from Calving to First Service (ICF): For multiparous cows, the interval in days between the calving date of the current parity and the first insemination date of the same parity. Insemination parities and calving parities were matched for each individual.

Calving Interval (CI): Interval in days between two consecutive calving dates of the same cow. Only individuals with confirmed breeding records (i.e., the last insemination record of the current parity) were included in the calculation.

Number of Services for Cows (NS_C): Total number of inseminations between the first and last insemination of the same parity for multiparous cows.

Interval from First to Last Inseminations in Cows (IFL_C): Interval in days between the first and last insemination of the same parity for multiparous cows.

A parity was considered valid if: (1) the last insemination was followed by a positive pregnancy diagnosis and a subsequent calving record; or (2) the interval between the subsequent calving date and the last insemination date was between 255 and 305 days.
